# Identification, Activity and Disulfide Connectivity of C-di-GMP Regulating Proteins in *Mycobacterium tuberculosis*

**DOI:** 10.1371/journal.pone.0015072

**Published:** 2010-11-30

**Authors:** Kajal Gupta, Prasun Kumar, Dipankar Chatterji

**Affiliations:** Molecular Biophysics Unit, Indian Institute of Science, Bangalore, India; University of Hyderabad, India

## Abstract

C-di-GMP, a bacterial second messenger plays a key role in survival and adaptation of bacteria under different environmental conditions. The level of c-di-GMP is regulated by two opposing activities, namely diguanylate cyclase (DGC) and phosphodiesterase (PDE-A) exhibited by GGDEF and EAL domain, respectively in the same protein. Previously, we reported a bifunctional GGDEF–EAL domain protein, MSDGC-1 from *Mycobacterium smegmatis* showing both these activities (Kumar and Chatterji, 2008). In this current report, we have identified and characterized the homologous protein from *Mycobacterium tuberculosis* (Rv 1354c) named as MtbDGC. MtbDGC is also a bifunctional protein, which can synthesize and degrade c-di-GMP *in vitro*. Further we expressed *Mtbdgc* in *M. smegmatis* and it was able to complement the MSDGC-1 knock out strain by restoring the long term survival of *M. smegmatis*. Another protein Rv 1357c, named as MtbPDE, is an EAL domain protein and degrades c-di-GMP to pGpG *in vitro*. Rv1354c and 1357c have seven cysteine amino acids in their sequence, distributed along the full length of the protein. Disulfide bonds play an important role in stabilizing protein structure and regulating protein function. By proteolytic digestion and mass spectrometric analysis of MtbDGC, connectivity between cysteine pairs Cys^94^-Cys^584^, Cys^2^-Cys^479^ and Cys^429^-Cys^614^ was determined, whereas the third cysteine (Cys^406^) from N terminal was found to be free in MtbDGC protein, which was further confirmed by alkylation with iodoacetamide labeling. Bioinformatics modeling investigations also supported the pattern of disulfide connectivity obtained by Mass spectrometric analysis. Cys^406^ was mutated to serine by site directed mutagenesis and the mutant MtbC406S was not found to be active and was not able to synthesize or degrade c-di-GMP. The disulfide connectivity established here would help further in understanding the structure – function relationship in MtbDGC.

## Introduction

The cell-cell communication or quorum sensing plays a major role in survival and maintenance of bacteria during the stationary phase. One of the interesting aspects of quorum sensing is the coordinated response of bacteria like biofilm formation, antibiotic production, sporulation, expression of virulence factor etc. [1]. A cell produces small autoinducer molecule and simultaneously senses the concentration of the autoinducer in the cell surface [2]. Second messengers act as autoinducers and relay signals received at the cell surface to target molecules within the cells. Nucleotide derivatives, which act as second messengers have been extensively studied for their regulatory function [3]. Cyclic adenosine monophosphate (cAMP), Cyclic guanosine monophosphate (cGMP), Guanosine 3′,5′ (bis) pyrophosphate (ppGpp) are all important second messengers both in prokaryotes and eukaryotes. cGMP is commonly used in eukaryotes but has very little role in bacteria [4]. cAMP is known to activate catabolite regulatory protein (CRP), a transcription regulator of gene involved in carbon metabolism [1], [5]. ppGpp on the other hand, regulates bacterial survival during nutrient starvation [6]. Another nucleotide, Bis-(3′-5′)-cyclic dimeric guanosine monophosphate (C-di-GMP) has been found to be involved in modulating cell surface and biofilm formation in several bacteria. This molecule was first reported more than 20 years back as a positive allosteric regulator of cellulose synthesis [7], [8].

C-di-GMP is synthesized from cyclization of two GTP molecules by diguanylate cyclase (DGC) and degraded to linear diguanylic acid (pGpG) by phosphodiestrases (PDE) [9], [10]. These two opposing enzymatic activities regulate the cellular pool of c-di-GMP. The DGC and PDE activity are encoded by conserved amino acid motifs GGDEF and EAL or HD-GYP, respectively [3], [11], [12]. With the advent of whole genome sequencing it is observed that GGDEF and EAL domain are ubiquitously present in all bacteria but absent in eukaryote [13]. Gram negative bacterial genomes harbor large number of proteins belonging to GGDEF-EAL domain super family, whereas Gram positive bacteria have small number of them. For example *Vibrio cholerae* and *Escherichia coli* has 53 & 36 proteins but *Bacillus subtilis* and *Mycobacterium smegmatis* has 7 & 1 GGDEF-EAL domain proteins, respectively [14]. In many cases GGDEF and EAL domains are present in tandem and most of the proteins so far characterized have either DGC or PDE-A activity. Interestingly, the possibility of opposing enzymatic activities co-existing in a single protein has also been reported [15], [16]. Our last report on MSDGC-1 from *M. smegmatis* was one such example of a bifunctional protein [17].

C-di-GMP has been implicated in regulation of many cellular responses relevant to pathogenesis, such as motility, secretion, cytotoxicity and biofilm formation. Most of the studies have indicated that the higher cellular level of c-di-GMP increases the biofilm formation and promotes the sessile form of life whereas a low level promotes motility [4], [18], [19], [20]. The appealing aspect of c-di-GMP signaling is the regulation of virulence gene and the well studied example is *Vibrio cholerae*. The vieA, an EAL domain protein not only controls the host colonization but also regulates the Cholera toxin production [15]. Similar reports of c-di-GMP mediated expression of virulence genes and survival in host have been published for *Salmonella typhimurium* and *Pseudomonas aeruginosa* [21], [22]. Though most of the studies have been done in Gram negative bacteria, few studies in Gram positive bacteria indicate that c-di-GMP may not play an important role in their physiology [23], [24]. Contrary to this we have earlier established the presence and physiological relevance of c-di-GMP in *M. smegmatis*, a GC-rich Gram positive bacterium [17]. The pathogenic species of Mycobacterium genera *M. tuberculosis* causes tuberculosis and is one of the oldest diseases known to man. This work was the initial attempt to characterize the stress elements in *M. tuberculosis* and we were interested in following the role of cell-cell communication in this organism.

**Figure 1:**
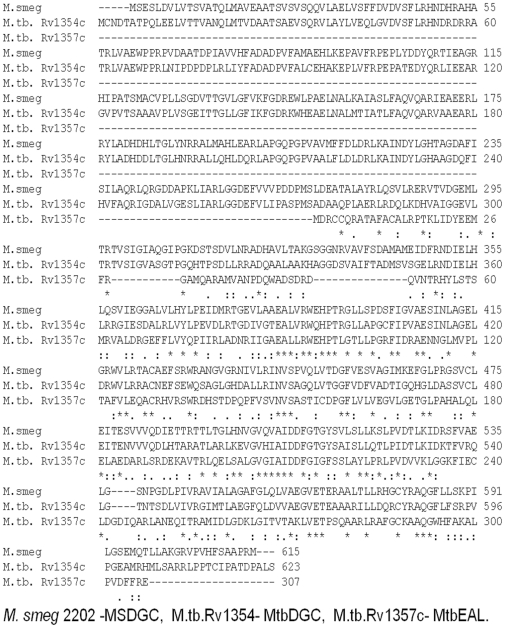
Sequence alignment of MSDGC-1, MtbDGC and MtbEAL was generated by Clustal W and was adjusted manually. Cysteines at the identical position in both the sequences are indicated by a black background.

Upon sequence alignment (Figure 1) we observed that MSDGC-1 showed 60% identity at amino acid level to MtbDGC, suggesting that the protein may have a similar role to play in *Mycobacterium tuberculosis.* Fig. 1 shows that three cysteine residues are conserved in MtbDGC and MSDGC-1, whereas only one cysteine residue is conserved in all the three proteins. Expression of MtbDGC in *E*. *coli* leads to production of the expressed proteins in insoluble inclusion bodies. IBs must be resolubilized and refolded into an active confirmation which requires extensive trial and error method. In this report we discuss the solubilization of the protein in its native form maintaining the c-di-GMP synthetic activity.

Disulfide bonds play an important role in stabilizing the native structure and regulating its basic function [25], [26]. Free cysteines residues are also important to establish protein function, ligand binding and catalysis [27]. Here we have used chemical and proteolytic cleavage of MtbDGC and identified the disulfide linkages by alkylation and Mass spectrometry [28], [29]. We observed that in MtbDGC the disulfide linkages exist between (Cys^94^-Cys^584^), (Cys^2^-Cys^479^) and (Cys^429^-Cys^614^). The Cys^406^ was found to be free and interestingly when mutated to serine the protein was found to be inactive. To further understand the structure and disulfide connectivity of MtbDGC protein, we report here bioinformatics modeling of MtbDGC protein.

## Materials and Methods

### Bacterial strains, plasmids and oligonucleotides

Bacterial strains, plasmids and oligonucleotides used in the study are listed in Table 1. DH5α and BL21 (DE3) *E. coli* strains were grown in LB broth at 37°C with agitation or on a plate containing 1.5% w/v agar. Antibiotics were used at following concentration as and when required: Ampicillin (100 µg ml^−1^) or kanamycin (35 µg ml^−1^) for *E. coli* and kanamycin (20 µg ml^−1^) or Hygromycin (20 µg ml^−1^) for *M. tuberculosis* or *M. smegmatis*. The PCR reactions were carried out using Dynazyme EXT polymerase. All of the clones generated were confirmed by sequencing (MWG, India). Restriction enzymes used for the cloning were procured from New England Biolabs or Fermentas.

**Table 1:** Bacterial strains plasmids and primers used in this study.

Strain, plasmid and primer	Characteristics	Source or Reference
Bacterial strains		
ΔMSDGC-1	*M. smegmatis* where MSDGC_2196 has been replaced with Kan cassette Kan^r^	(Kumar et al., 2008)
MtbRv1354 complimented	ΔMSDGC complemented with MtbDGC Kan^r^ and Hyg^r^	This work
pET MtbDGC	MtbRv1354c cloned in pET 21b Amp^r^	This work
pET MtbPDE	MtbRv1357c cloned in pET 21b Amp^r^	This work
Plasmids		
pET21b	Cloning vector (c-terminal His tagged) Amp^r^	Novagen
pMV361	Cloning vector with hsp60 promoter, kan^r^	(Stover et al., 1991)
Primers	Sequence (5′-3′)	
MtbRv1354f	AGCATCCCATATGTGCAACGACACCGCGAC	
MtbRv1354r	GCACCTCTCGAGAGATAACGCCGGGTCAG	
MtbRv1357f	ATGCAGCCCTCATATGGATCGTTGTTG	
MtbRv1357r	CACGCAAGCTTCTCTCTGAAAAAGTCG	
MtbC406F	CTGCTGGCACCGGGCTCCTTCATCCCTGTG	
MtbC406R	CACAGGGATGAAGGAGCCCGGTGCCAGCAG	

### Cloning of MtbDGC and MtbPDE

Genes Rv 1354c (1872bp, 623aa) and Rv 1357c (924bp, 307aa) were PCR amplified from genomic DNA of *M. tuberculosis H37Rv* using a set of primers, MtbRv1354f, MtbRv1354r, MtbRv1357cf and MtbRv1357cr respectively (Table 1). The amplicons Rv 1354 and Rv 1357c were cloned in pET21b vector using *NdeI - XhoI* and *NdeI - HindIII* restriction sites, respectively. The resultant plasmids were named as pETMtbDGC for Rv1354c and pETMtbPDE for Rv1357c.

### Expression, isolation and purification of MtbDGC and MtbPDE from inclusion bodies

BL-21 cells carrying MtbDGC and MtbPDE plasmids were grown at 37°C in 2 L flask containing 500 ml of LB medium supplemented with 100 µg/ml of ampicillin. Protein expression was induced when cell density was reached to 0.6 at OD_600_ with 1 mM of isopropyl-D-thiogalactopyranoside (IPTG). After 4 h induction, cells were harvested by centrifugation at 8,000 rpm for 15 min at 4°C. Cells were lysed in lysis buffer containing 50 mM Tris-HCl at pH 7.9, 500 mM NaCl, 100 mM Dithiothreitol (DTT), 1 mM Ethylenediaminetetraacetic acid (EDTA), 6 M Urea and 20% Glycerol. Cell suspensions were sonicated at 150 kHz, using a sonicator with a 13 mm probe. This cycle was repeated three times for a total sonication time of 5 min each. Cell debris was removed by centrifugation at 12,000 rpm for 20 min at 4°C. After centrifugation the supernatant was loaded on Nickel-Nitrilotriacetic acid (Ni-NTA) column and washed with 100 column volumes of wash buffer containing 10 mM imidazole. The protein was eluted with the elution buffer containing 500 mM imidazole. Eluted protein was further dialyzed as reported below.

### Refolding of MtbDGC and MtbPDE proteins with step-wise dialysis

The protein solubilized in the elution buffer containing 500 mM imidazole was subsequently dialyzed at various steps with decreasing concentration of urea in the buffer containing 50 mM Tris-HCl (pH 7.9 at 4°C), 250 mM NaCl, 10 mM DTT, 5% Glycerol. Urea concentration was varied from 6 M to 0 M and the dialysate was allowed to reach equilibrium [30]. As reported earlier disulfide containing proteins can refold even in the presence of concentrated denaturant [31]. At the final stage with no urea, precipitation of the protein was not observed. Finally the protein was dialysed against the same buffer having no DTT and assayed for its activity.

### Enzymatic assays

Di-guanylate cyclase and phosphodiesterase assays were adapted from procedures described previously [17], [32]. Both the activities were followed in a buffer containing 5 µM of protein, MtbDGC, MtbPDE or MSDGC-1 and 25 mM Tris-HCl (pH 7.9), 250 mM NaCl, 10 mM MgCl_2_ in 50 µl volume. The reaction was triggered by the addition of a mixture of 0.1 mM Guanosine 5′-triphosphate (GTP) and α-labeled [^32^P]GTP (Board of Radiation and Isotope Technology (BRIT), India; 0.01 µCi µl^−1^ or 3500Ci mmol^−1^) in the case of MtbDGC. However, MtbPDE activity was checked by replacing GTP with c-di-GMP. Aliquots were withdrawn at regular time intervals and the reaction was stopped with an equal volume of 50 mM EDTA. Reaction products (2.5 µL) were separated on polyethyleneimine-cellulose plates (Merk) in 1∶1.5 (v/v) saturated NH_4_SO_4_ and 1.5 M KH_2_PO_4_ (pH 3.6) and plates were exposed to a phosphor-imager screen. The [^32^P] c-di-GMP, prepared as described below was used as substrate for PDE-A activity. Preparation of [^32^P] labelled and nonlablled c-di-GMP was done by the protocol as described earlier [10].

### Western blot analysis

Western blot analysis for the detection of MtbDGC was carried out with primary antibodies raised in rabbit against the purified protein. Total cellular proteins extracted from cells grown at different time were normalized and 100 µg of the lysate was separated by Poly acrylamide gel electrophoresis (SDS-PAGE) and blotted on to a Sodium Dodecyl Sulphate -Polyvinylidende Fluoride (PVDF) membrane. The polyclonal serum generated was used as primary antibodies and a secondary antibody was purchased from Sigma Aldrich. Both were used after ten thousand fold dilution. The blots were developed with 26 mg aminoethylcarbazole ml^−1^ and 0.01% v/v H_2_O_2_.

### Long term starvation cultures

The ΔMSDGC-1, wild type strain of *M. smegmatis* mc^2^ 155 and strain developed by complementing ΔMSDGC-1 with *Mtbdgc* were grown in MB7H9 with 0.02% w/v glucose and 0.05% v/v Tween−80 till saturation. Cultures were declumped before plating on agar described earlier [29]. The colony forming units of these cultures were determined at regular interval of time up to 20 days. Antibiotics were omitted from the culture to rule out any possible effect of antibiotics on long term survival.

### Proteolytic digestion of protein (MtbDGC)

10 µg of purified full length protein was diluted to final acetonitrile concentration of 5–10% in presence of digestion buffer (50 mM of ammonium bicarbonate) and subsequently digested at 37°C overnight using the following proteolytic enzymes in individual experiments. MtbDGC was digested with 200 ng trypsin (sequencing grade, modified trypsin (promega)), 200 ng chymoptrypsin (sequencing grade, modified trypsin (promega)) and double digested with 100 ng of trypsin and 100 ng of chymotrypsin respectively. The proteolytic digestion mixtures were desalted using C-18 column (Supelco) and subsequently analyzed by Matrix-Assisted Laser Desorption Ionization Mass Spectrometry (MALDI MS) and (Liquid Chromatography-Electrospray Ionisation) LC-ESI-MS/MS.

### Reduction and alkylation of digested protein sample

10 µL of purified protein was taken in 15 µL of 0.1 M ammonium bicarbonate buffer pH 8.0 for alkylation. For the reduction, DTT was added to a final concentration of 8 mM and was incubated at 37°C for 3 h. For alkylation iodoacetamide (IAM) was added to the final concentration of 40 mM. The mixture was incubated at room temperature in the dark for 90 mins. Reaction mixture was incubated at 37°C overnight for further digestion. Two sets of reactions were carried out, one to check for alkylation and the others for reduction and followed by alkylation.

### MALDI-TOF Mass spectrometry

Mass-spectra of digested gel spots were obtained by Matrix-Assisted Laser Desorption Ionization time of flight (MALDI-TOF) mass spectrometry on an Ultraflex TOF/TOF spectrometer (Bruker Daltonics). All the mass spectra were acquired in positive-ionization mode with reflectron optics. The instrument was equipped with 50 Hz pulsed nitrogen laser (λ = 337 nm) and operated under delayed extraction conditions; delayed time 90-ns, and 25 kV accelerating voltage. All peptides samples were prepared by mixing an equal amount of matrices dihydroxybenozic acid/α-cyano-4-hydroxycinnamic acid saturated in 0.1% Trifluoroacetic acid and acetonitrile (1∶1). Typically, 50–100 laser shots were used to record each spectrum.

### Tandem Mass Spectrometry (LC-ESI-MS, MS/MS)

For all the disulfide connectivity studies, the proteolytic peptide mixture was analyzed by reverse-phase HPLC-ESI-MS, MS/MS (Electrospray Ionisation, ESI, Bruker Daltonics) spectrometry. Peptides were separated on HPLC (1100 series HPLC (Agilent)) system equipped with C18 column (Supelco). The column eluant was directly coupled to a HCT Ultra PTM Discovery System (ETD II- Bruker Daltonics). Mass spectra (ESI-MS) and tandem mass spectra (ESI-MS/MS) mode were recorded in positive-ion mode with a resolution of 12,000-15,000 Full Width of Half Maximum (FWHM). For tandem mass spectrometry dissociation, the mass analyzer was set to ±1 *m/z*. The precursor ions were fragmented in a collision cell using nitrogen as the collision gas. Spectra were calibrated in static mode using MS/MS fragment ions of standard supplied by the manufacturer. The enzymatically digested peptides were separated using mobile phase A consisted of 0.05% formic acid in 98% H_2_O/2% CAN (Acetonitrile) and B consisted of 0.05% formic acid in 98% ACN/2% H_2_O with a gradient of 5% B in first 5 mins, followed by 95% B in next 50 mins and 5% B in last 10 mins. The gradient times were varied depending upon the sample specifications.

### Homology modeling, minimization and model validation

The sequence and domain information of 623 amino acid length Rv1354c was taken from swissprot (ID:P64826; http://us.expasy.org/sprot/) [33]. Protein consists of three domains named as GAF (residue 28-171), GGDEF (residue 212–345) and EAL (residue 354–609). Since no homology was found for the full length protein, Modeling of individual domain was done and joined to give the complete structure. BLAST [34] and PSI-BLAST [35] was used against National centre for biotechnology information (NCBI), Protein Data Bandk (PDB) database to find top hit as a template and target vs template sequence alignment for individual domains was done using CLUSTALW2 [36]. On the basis of sequential similarity 10 models each of EAL and GGDEF domain were modeled with the protein PDB [37] entry 2R6O (chain:A;identity:40%) and PDB entry 1W25 (chain:A;identity:40%) respectively using MODELLER release 9v7 [38] and model using PHYRE [39] server. GAF and EAL domain consists of N terminal (residue 1–27) and C terminal (residue 610–623) of full protein respectively. Considering three modeled domains as template full with the minimum DOPE score was selected. A threaded model of GAF domain was produced convergence to machine precision. Minimized structure was validated with the programs PROCHECK score (91.8%) [40], VERIFY3D (78.04%) [41] and PROSA score (−6.68) [42]. PyMOL [43] was used for 3D-structure visualization, protein was modeled using MODELLER 9v7. Top scored model was solvated in SPC216 water box (total number of water molecule: 93396) and charge neutralization was accomplished with the addition of 7 Na+. Energy minimization of the model was carried out by GROMACS 3.3.1(The Gronian Machine for Chemical Simulations) [44] using OPLS [45] force field and steepest descent method. The minimization was set to run for 10000 steps with 0.002 ps time step or until.

### Alignment of proteins containing GAF, GGDEF, EAL architecture

Since the protein of interest was having three domains namely GAF, GGDEF, & EAL domain, protein sequences with the above architecture were searched in PFAM database [46]. A total of 183 sequences were found. Many of the protein sequences consisted of transmembrane or low complexity regions and there was a large variation in length among the sequences. This variation was more prominent in the region before GAF domain and the relative positioning of each domain with respect to other. Linker region between the GAF and GGDEF domain also varied in length. Each domain was separated from the corresponding sequences by taking the domain information given in the PFAM database as SWISSPROT did not provide the domain start & end information for all 183 sequences. Alignment of each of the domain sequences was done separately using ClustalW2. Although there was ambiguity in the assignment of start and end positions of each domain of proteins in PFAM and SWISSPROT databases, the SWISSPROT data was considered for modelling as it is supported by experimental methods.

### Cysteine mutation using site directed mutagenesis

Third cysteine of MtbDGC at position 406 was mutated to serine amino acid using PCR based approaches. In this method two complimentary oligonucleotides containing the desired mutation were used to prime the PCR on a pET MtbDGC plasmid DNA template. Conditions used for PCR are as follows - in total volume of 50 µL template DNA 100 ng, thermostable polymerase buffer (10×) 5 uL, mutagenic primer 20 pmol, 0.6 µL of 25 mM dNTPs solution and thermostable polymerase 2–5 U. The tube was initially heated at 94°C for 2 min, followed by 19 cycles of 94°C for1 min, for annealing 54°C for 1 min and an extension step at 72°C for 8 mins, the tube is held at 72°C for 20 mins and if required at 4°C indefinitely. Subsequently digestion of the reaction mixture with *DpnI* was done to removes the template DNA leaving intact newly synthesized double stranded mutant PCR product designated as MtbC406S [47], [48]. The primers used in mutagenesis are listed in Table 1. The clone was confirmed by sequencing at MWG (data not shown), Bangalore. The plasmid MtbC406S was further transformed in BL-21 *Escherichia coli* cells. Protein was expressed and purified as described in section 2.3. Activity of the mutated protein was checked as described in section 2.5.

### Circular dichorism study of MtbDGC and MtbC406S

The purified MtbDGC and MtbC406S protein after folding was further dialyzed against the CD buffer containing 10 mM phosphate buffer pH 8.0, 150 mM KCl and was filtered through a 0.2 µM PVDF filter (Milipore). 5 uM of MtbDGC and MtbC406S protein were used for CD studies. Circular dichorism spectra were recorded on a Jasco J-715 spectopolarimeter at 22°C using a cell of path - length 0.2 cm, bandwith 2.0 nm and a response time of 2s. Spectra were averaged over three scans with each sample and were recorded at a speed of 50 nm/min from 250 nm to 200 nm in a quartz cuvette.

## Results and Discussion

### MtbDGC possesses both DGC and PDE-A and MtbPDE possesses PDE-A activity

**Figure 2:**
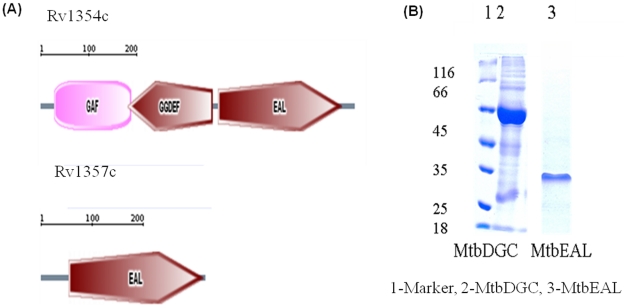
Representatives of C-di-GMP specific domain architectures in *Mycobacteirum tuberculosis* and their subsequent protein purification. (A) Domain architecture of the protein with GGDEF and EAL domain in *Mycobacterium tuberculosis*. The length of each sequence is approximately to scale. (B) Purification of C- terminal His_6_ tag proteins Lane – 1 Marker, Lane - 2 MtbDGC and Lane – 3 MtbPDE. C-terminal His_6_ –tagged Rv1354 is 67.6 KDa and Rv1357c is 33.9 KDa. Purity of the preparations was determined by (10%) SDS- PAGE.

**Figure 3:**
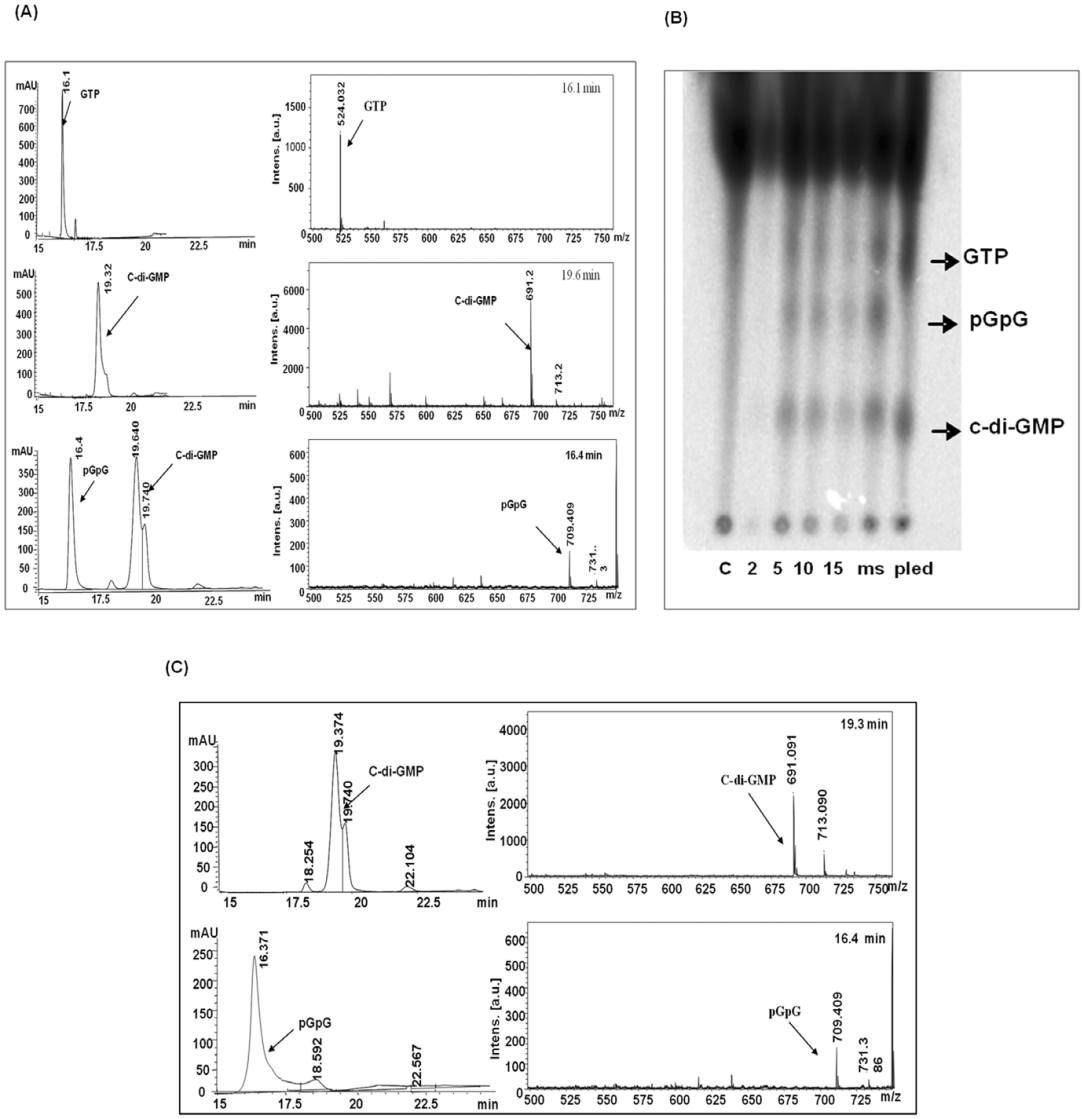
MtbDGC is a bifunctional protein showing both diguanylate cyclase (DGC) and phosphodiesterae acitivity (PDE-A) and MtbPDE is a functional protein showing phosphodiesterae acitivity (PDE-A). (A) Purified His_6_ protein was assayed for the ability to synthesize c-di-GMP. HPLC analysis for the detection of c-di-GMP and pGpG: (a): GTP, (b): Purified c-di-GMP, (c): With protein MtbDGC, separated on reversed phase HPLC. MALDI TOF analysis of the relevant HPLC fractions, in the positive ion detection mode, ions were detected at m/z of 542 (M+H)^+^ for GTP, 691 (M+H)^+^ and 713 (M+Na)^+^ for c-di-GMP, 709 (M+H)^+^ and 731 (M + Na)^+^ for pGpG. (B) Purified His_6_ protein was assayed for the ability to degrade c-di-GMP. HPLC analysis for the detection of pGpG. (c): Purified c-di-GMP (C): With Protein (MtbPDE),. MALDI TOF analysis of the relevant HPLC fractions, in the positive ion detection mode, ions were detected at m/z  = 691 (M+H)^+^ and 713 (M+Na)^+^ for c-di-GMP, 709 (M+H)^+^ and 731 (M + Na)^+^ for pGpG.

Domain organization of Rv 1354c and Rv 1357c was obtained from SMART database as shown (Figure 2A). Rv 1354c and Rv 1357c are named and referred throughout the manuscript as MtbDGC and MtbPDE, respectively. MtbDGC and MtbPDE were purified in soluble form as described in materials and methods to homogeneity as judged by SDS-PAGE profile (Figure 2B). Both the proteins were more than 80% pure (Protein id. NP-215870.1). Further digestion of the band and subsequent MALDI and MASCOT analysis confirmed the presence of MtbDGC and MtbEAL proteins (data not shown). These protein preparations were used to check the DGC and PDE-A activity. The reaction products were separated on reverse phase High performance liquid chromatography (HPLC) along with GTP and purified c-di-GMP as controls. Further, these peaks were collected and subjected to MALDI-TOF analysis. The major ions detected were *m/z* 542 (M+H)^+^ for GTP, 691 (M+H)^+^ and 713 (M+Na)^+^ for c-di-GMP, 709 (M+H)^+^ and 731 (M + Na)^+^ for pGpG, thus confirming that MtbDGC is capable of both synthesizing and degrading c-di-GMP (Figure 3A). Reaction mixture with MSDGC-1 protein (Kumar and Chatterji, 2008), a known DGC protein of *Mycobacterium smegmatis* was used as control. On the Thin Layer Chromatography (TLC) plate the spot at the same Rf of c-di-GMP apparently followed by another spot with Rf of pGpG. Reaction mixture with MSDGC-1 was used as a positive control and reaction mixture with cell lysate was used as the negative control (Figure 3B).

Similarly, the reaction mixture where c-di-GMP was incubated with MtbPDE showed reduction in peak area of c-di-GMP and appearance of an additional peak at 16.4 min at the same retention time of pGpG. The mass spectrum of 16.4 min peak showed major ion at *m/z* 709, an addition of a water molecule to c-di-GMP (Figure 3C). The purified pGpG showed the identical mass spectrum at *m/z* 709 confirming that the MtbPDE possesses phosphodiesterase activity.

### MtbDGC is functional *in vivo*

**Figure 4:**
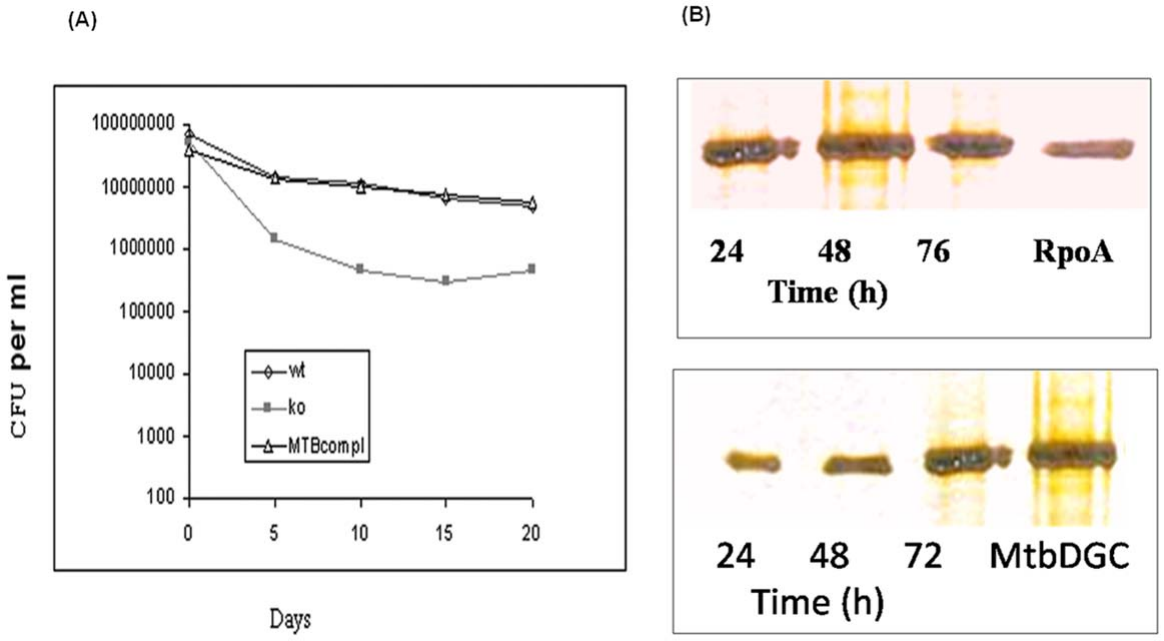
Complementation of ΔMSDGC-1 with MtbDGC and its long term survival. (A) ΔMSDGC-1 a strain of *M. smegmatis* was complemented with MtbDGC and its growth was compared with wildtype as well as ΔMSDGC-1. Cultures were grown in MB7H9 medium with 0.02% v/v glucose and 0.05% Tween-80 as carbon source. Aliquots were withdrawn at different time interval after culture reached stationary phase (48 hours of growth) and plated on MB7H9 agar supplemented with Kanamycin containing 2% w/v glucose as carbon source. The colony forming unit were determined and plotted. (B) Level of MtbDGC increases in stationary phase. ΔMSDGC-1 was transformed with MtbDGC and expression was studied in three phases of growth i.e. exponential, early stationary and late stationary phase. The α subunit (RpoA) of RNA polymerase was used as an control because its level remains unchanged in different phases of growth. Whole cell lysates was separated on 10% SDS-PAGE and analyzed by Western blot technique probed with antibody raised against MtbDGC.

Previously we reported that the MSDGC-1 which possesses both DGC and PDE-A activities is required for long term survival in *M. smegmatis*, under nutritional starvation. MtbDGC is also bifunctional and homologous to MSDGC-1. It is intriguing to test the *in vivo* functionality of MtbDGC in the MSDGC-1 disrupted mutant ΔMSDGC-1. We complimented this mutant through chromosomal integration of MtbDGC in ΔMSDGC-1 strain using pMV361 integration vector containing Rv1354c gene. These strains were grown in carbon starved conditions and their survival was followed in terms of CFU until 20 days of incubation. Figure 4A shows that MtbDGC was able to restore the long term survival phenotype of the mutant strain as the CFU of wild type and complimented strain was comparable.

### Level of MtbDGC increases in stationary phase of growth

As described earlier, the level of MtbDGC in bacteria varies according to the environmental change and the growth phase. We determined the level of MtbDGC expression in three phases of the growth, i.e. exponential, early stationary and late stationary phase. Whole cell lysate of *M. tuberculosis* compliment was separated on SDS-PAGE and analyzed by Western blot technique with polyclonal antibodies raised in rabbit against MtbDGC protein. An immunoreactive protein band, approximately 68 kDa in size (Figure 4B) was detected, with increased band intensity in the stationary phase. Figure 4B shows that the amount of MtbDGC was three times higher in the stationary phase culture than in the exponential phase, as detected by quantization of the blot. In each lane, the same amount of protein (100 µg) was loaded. It should be mentioned here that the growth of the bacteria was found to be slow when the *dgc* gene was over expressed (data not shown).

### Proteolytic digestion of MtbDGC protein

**Figure 5:**
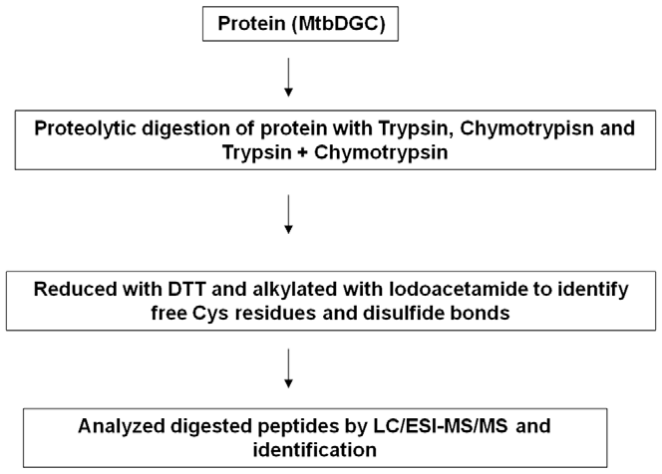
Schematic diagram of experimental setup to study disulfide connectivity pattern.

The flow chart for the protein digestion is depicted in Figure 5. Protein was digested with trypsin, chymotrypsin and in combination of both in three different sets of experiments. The ratio of concentration of proteolytic enzyme (Trypsin and Chymotrypsin) to protein affects the LC-ESI-MS/MS response. The optimal ESI signal was obtained when the ratio of enzyme to protein was 1∶ 20. Injection of 10–15 nmol of digested protein into the capillary of LC-ESI-MS/MS system produced a signal sufficient to characterize peptide containing labeled Cys and disulfide bonded Cys residues (data not shown).

### Mass spectral characterization of free cysteine residue and disulfide bonds

**Figure 6:**
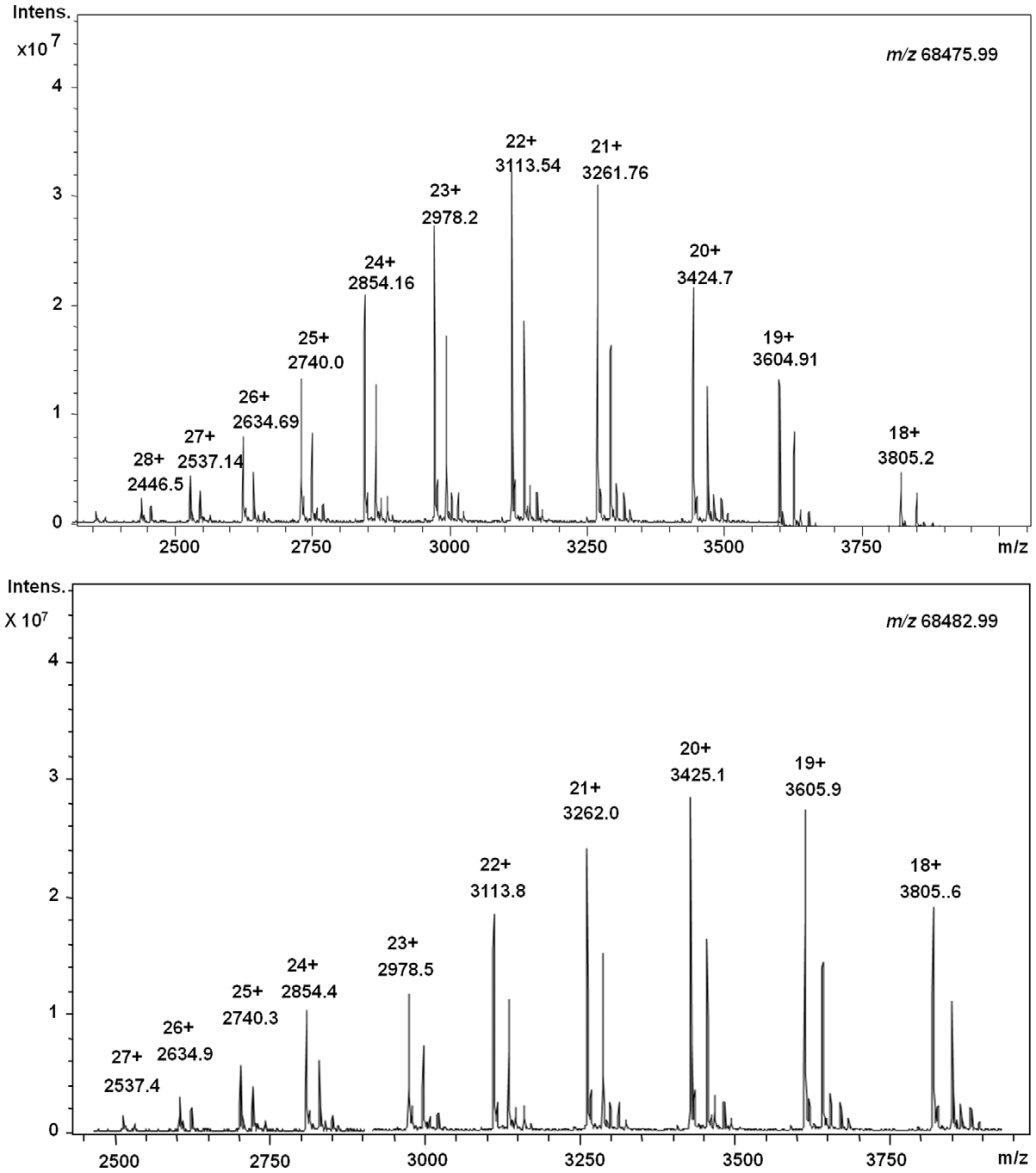
LC-ESI-MS of intact MtbDGC protein. (A) LC-ESI-MS of Histidine tagged intact MtbDGC protein and corresponding inset zoom of deconvoluted mass *m/z* 68475.99 of the protein. (B) LC-ESI-MS of DTT reduced MtbDGC intact protein and corresponding inset zoom of deconvoluted mass (*m/z* 68482.99). The shift in seven Dalton was observed in comparison of unreduced protein.

In order to identify number of disulfide bonds and free cysteine in the intact MtbDGC protein, LC-ESI-MS of intact protein was studied, which confirmed *m/z* of 68475.99 (His_6_ protein). The protein was further reduced with 8 mM DTT and mass difference of seven Daltons *m/z* 68482.99 (His_6_ protein) was obtained confirming the presence of at least three disulfide bonds in the protein (Figure 6A and 6B).

**Figure 7:**
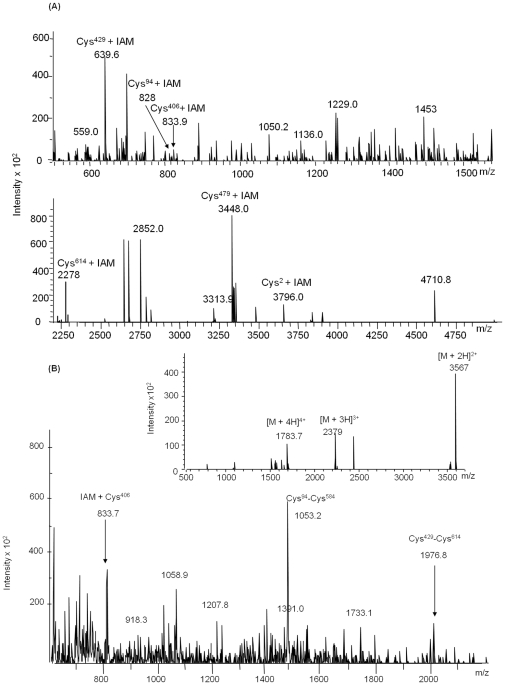
LC-ESI-MS of MtbDGC in reduced and oxidized condition, after digestion with chymotrypsin and trypsin. (A). LC-ESI-MS of DTT reduced and iodoacetamide alkylated MtbDGC digested with trypsin and chymotrypsin. The singly charged ions at *m/z* 3796.2, 828, 833.7, 639.6, 3448.0 and 2775.0 show the mass of cysteine peptides at Cys^2^, Cys^94^, Cys^406^, Cys^429^, Cys^479^ and Cys^614^ respectively (B) LC-ESI-MS of unreduced and iodoacetamide alkylated MtbDGC digested with trypsin and chymotrypsin. The singly charged ions at *m/z* 833.7, 1053.2 and 1399.5 [M +2H]^2+^ show the free Cys^405^ residue at third position and disulfide bond at Cys^94^-Cys^584^ and Cys^429^-Cys^614^ position respectively. Corresponding insect zoom shows the disulfide bonded Cys^2^-Cys^478^, the dominant ion is the doubly charged ion of [M +2H]^2+^ at *m/z* 3566.5.

A common problem in detecting peptides which contain free cysteine residues is that the sulfhydryl group reacts with other chemical in the solution. Thus, the cysteine residues in the protein are alkylated by alkylting reagent like iodoacetamide, iodoacetic acid or 4-venyl pyridiene. Here, we used iodoacetamide to alkylate the samples, using five fold molar excess of iodoacetamide, and we were able to detect all seven cysteine containing peptides in proteolytic digested protein in MALDI as well as by LC-ESI-MS analysis. A sample that displayed an isotopic distribution of the required peptides was further used for the studies. Figure 7(A) shows a representative mass spectrum from DTT (200-fold molar excess) reduced and iodoacetamide labeled protein after digestion with a combination of trypsin and chymotrypsin. Ions at *m/z* 3796.2 (aa. 1–35), 828 (aa. 92–98), 833.9 (aa. 400–407), 693.6 (aa. 428–432), 3448.8 (aa. 461–494), 341.3 (aa. 584–585) and 1452.6 (aa 610–623) represents Cys^2^, Cys^94^, Cys^406^, Cys^429^, Cys^479^ containing peptides, respectively, matching with the calculated value as depicted in table 2. Figure 7(A) was compared with mass spectrum depicted in Figure 7(B) generated by iodoacetamide labeled, oxidized protein after double digestion with trypsin and chymotrypsin. Singly charged [M + H]^+^ 833.7, 1053.2 and doubly charged [M +2H]^2+^ 3566.5 and 1399.5 ions corresponds to the calculated mass of free cysteine peptide Cys^406^ and disulfide connected dipeptide Cys^94^-Cys^584^, Cys^2^-Cys^479^ and Cys^429^-Cys^614^, respectively, as depicted in table 3. Each fragment that contained iodoacetamide labeled Cys^406^ residue showed a mass shift of 57 Da (Mass of iodoacetamide) compared with the mass of unlabelled peptide.

**Table 2:** Theoretically and experimental mass of peptides generated from reduced, alkylated and digested samples of MtbDGC.

Position of Cysteine in Protein	Expected Mass(m/z)		Observed Mass(m/z)	
	Unlabelled	Labeled	Unlabelled	Labeled
**Digested by Trypsin + Chymotrypsin**				
Cys^2^ (aa- 1-35)	3739.2	3796.2	3739.2	3796.2
Cys^94^ (aa-92-98)	770.9	827.9	770.9	828.9
Cys^406^ (aa-400-407)	776.9	833.9	776.9	833.7
Cys^429^ (aa-428-432)	582.6	639.6	582.6	639.6
Cys^479^ (aa-461-494)	3391.8	3449.8	3391.8	3448.0
Cys^584^ (aa-584-586)	284.3	341.3	284.3	341.3
Cys^614^ (aa-610-629)	2218.51	2275.51	2218.0	2275.0
**Digested by Chymotrypsin**				
Cys^2^ (aa-1-39)	4185.7	4242.7	4185.7	4242.0
Cys^94^ (aa-92-112)	1257.4	1313.4	1256.0	1313.4
Cys^406^ (aa-395-407)	1396.6	1453.4	1396.0	1453.0
Cys^429^ (aa-424-432)	1107.3	1164.3	1107.3	1164.0
Cys^479^ (aa-461-513)	5342.0	5399.0	5342.0	5399.0
Cys^584^ (aa-562-590)	2591.9	2648.9	2591.9	2648.6
Cys^614^ (aa-594-629)	4151.8	4208.8	4151.8	4208.0
**Digested by Trypsin**				
Cys^2^ (aa-1-35)	3739.2	3796.1	3739.6	3796.6
Cys^94^ (aa-81-98)	2023.3	2080.3	2025.1	2097.8
Cys^406^ (aa-400-422)	2355.7	2412.7	2353.0	2410.0
Cys^429^ (aa-423-427)	2204.4	2261.4	2201.0	2258.0
Cys^479^ (aa-448-494)	4997.5	5054.5	4998.0	5559.0
Cys^584^ (aa-584-586)	440.5	497.52	441.2	498.2
Cys^614^ (aa-610-629)	2218.5	2275.5	2218.5	2275.5

**Table 3:** Theoretically and experimental mass of peptides generated from intact proteins samples of MtbDGC upon oxidation.

Position of Cysteine in Protein	Expected Mass(m/z)		Observed Mass(m/z)	
	Unlabelled	Labeled	Unlabelled	Labeled
**Digested by Trypsin + Chymotrypsin**				
(Cys^2^ - Cys^479^)	7129.0[(3739.2+3391.8) -2 = 7129.0]	7129.0	[M+2H]^2+^: 3566.5	[M+2H]^2+^: 3566.5
(Cys^94^ - Cys^584^)	1053.2[(770.9+284.3) -2 = 1053.2]	1053.2	[M+H]^+^: 1053.2	[M+H]^+^: 1053.2
Cys^406^	776.9	833.9	[M+H]^+^776.9	[M+H]^+^: 833.7
(Cys^429^- Cys^614^)	2799.1[(582.6+2218.5) -2 = 2799.1]	2856.1	[M+H]^+^: 2799.1	[M+H]^+^: 2856.0
**Digested by Chymotrypsin**				
(Cys^2^ - Cys^479^)	9525.7[(4185.7+5342) – 2 = 9525.7]	9525.7	[M+3H]^3+^3176.9	[M+3H]^3+^3177.9
(Cys^94^ - Cys^584^)	3847.9[(1257.4+2591.9) – 2 = 3847.9]	3847.9	[M+H]^+^: 3846.2	[M+H]^+^: 3846.2
Cys^406^	1396.6	1453.6	[M+H]^+^1396.0	[M+H]^+^: 1453.4
(Cys^429^- Cys^614^)	5259.1[(1107.3+4151.8) – 2 = 4434.2]	5316.1	[M+H]^+^2629.5	[M+H]^+^: 2629.5
**Digested by Trypsin**				
(Cys^2^ - Cys^479^)	9145.0[(3739.2+4997.7) – 2 = 8734.7]	8791.7	[M+3H]^3+^: 2911.5	[M+3H]^3+^: 2911.5
(Cys^94^ - Cys^584^)	244384.0[(2202+2184) – 2 = 4384.0]	4384.0	[M+2H]^2+^: 2461.4	[M+2H]^2+^2461.4
Cys^406^	2355.7	2412.7	2353.0	2410.0
(Cys^429^- Cys^614^)	4420.9[(2204.4+2218.5) – 2 = 4420.9]	4477.9	[M+H]^+^:4420.9	[M+H]^+^4477.9

To evaluate and confirm the pattern obtained from trypsin and chymotrypisn treated double digested protein, MtbDGC protein was also digested with chymotrypsin or trypsin. The *m/z* 4242.7 (aa. 1–39), 1313 (aa. 92–112), 1453.0 (aa. 395–407), 1164.0 (aa. 424–432), 5399.0 (aa. 461–513), 2648.9 (aa. 562–590) and (His_6_) 3385.2 (aa. 594–629) ions are in good agreement with the calculated mass of free cysteine containing peptide labeled with iodoacetamide (Table 2) when digested with Chymotrypsin (data not shown). The predicted disulfide linked peptides Cys^429^-Cys^614^, Cys^94^-Cys^584^ and Cys^2^-Cys^479^ were also detected in chymotrypsin digested and reduced protein. The *m/z* for 3176.9 [M +3H]^3+^, 3846.2, 4491.6 [M + H]^H+^ (table 3) ions represents dipeptide linked Cys^2^-Cys^479^, Cys^94^-Cys^584^ and Cys^429^-Cys^614^ respectively. The *m/z* 1453.0 for the [M + H]^+^ ion shows free cysteine at Cys^406^. Similar results were also obtained with trypsin digested protein (data not shown).

### Disulfide connectivity

Disulfide connected dipeptide were analyzed by LC-ESI-MS/MS and individual peptides were identified based on the information obtained by tandem mass spectra. The technique involves the breakage of peptide backbone generating predominantly b and y-type ions [49]. The LC-ESI-MS/MS has been shown to give ambiguous result due to thiol exchange reaction. To overcome this problem all the analysis was done in the presence of alkylting agent iodoacetamide. To prove that the disulfide bonding is not altered, alkylation was done before or after protein was proteolyticaly digested.

**Figure 8:**
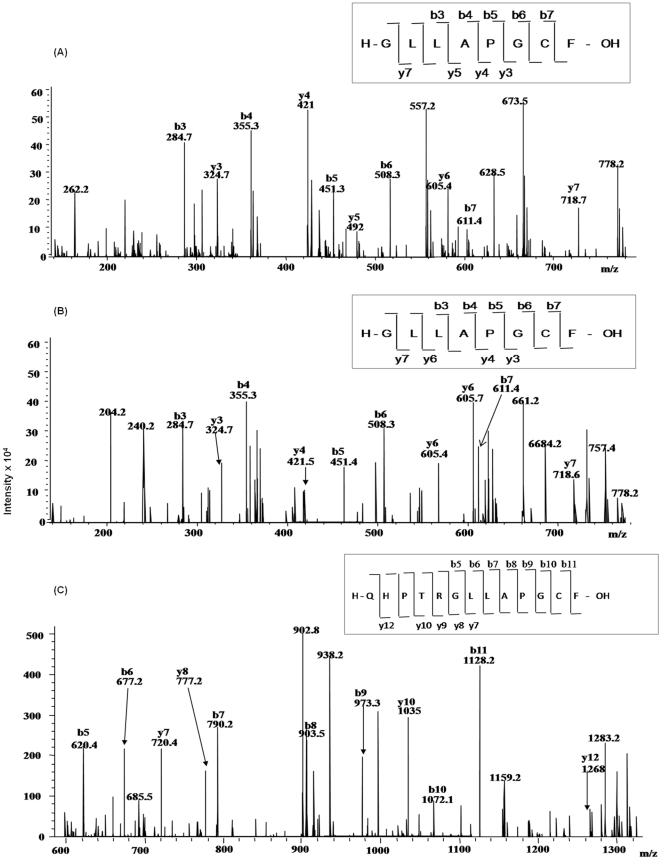
LC-ESI-MS/MS of peptide containing free Cysteine406. (A). LC-ESI-MS/MS of *m/z* 776.6, containing free Cys^406^ from DTT treated, trypsin and chymotrypsin digested MtbDGC. Inset shows the sequence derived from this MS/MS spectrum. (B) LC-ESI-MS/MS of *m/z* 833.7, containing free Cys^406^ from unreduced trypsin and chymotrypsin digested MtbDGC. Inset shows the sequence derived from this MS/MS spectrum. (C) LC-ESI-MS/MS of *m/z* 1396, containing free Cys^406^ from DTT treated, chymotrypsin digested MtbDGC. Inset shows the sequence derived from this MS/MS spectrum.

MS/MS analysis of singly charged [M + H]^+^ ions at *m/z* 776.6 (aa. 400–407) obtained from trypsin and chymotrypsin digested protein after DTT treatment confirmed Cys^406^ as a free cysteine containing peptide, Figure 8A. The Collision induced dissociation (CID) spectrum of the linearized peptide showed successive b and y-ions of *m/z* 284.7 (b3), 355 (b4), 451.3 (b5), 508.3 (b6), 611.4 (b7), 324.7(y3), 421 (y4), 492 (y5) and 718.7 (y7). The mass difference between the successive b or y ions represented the residual mass of amino acid as obtained by theoretical digestion. More than 50% of the predicted amino acid sequence obtained by MS/MS of the peptide matched with Mascot search data base. Figure 8B represents the free Cys^406^ peptide ion *m/z* 833.7 obtained from chymotrypsin and trypsin digested and iodoacetamide labeled protein in unreduced condition. The mass differences between the successive b or y ions are in agreement with the calculated mass. To further confirm that Cys^406^ is free in MtbDGC protein was digested with chymotrypsin in oxidized condition and was analyzed by LC/ESI/MS-MS. Singly charged [M +H]^+^ ion at *m/z* 1396.0 confirmed peptide containing Cys^406^. MS/MS of unreduced, trypsin digested and iodoacetamide labeled sample also confirmed Cys^406^ to be a peptide containing free cysteine (Figure 8C). Spectra were also acquired to locate free cysteine in reduced condition for trypsin and Chymotrypsin digested protein (data not shown).

**Figure 9:**
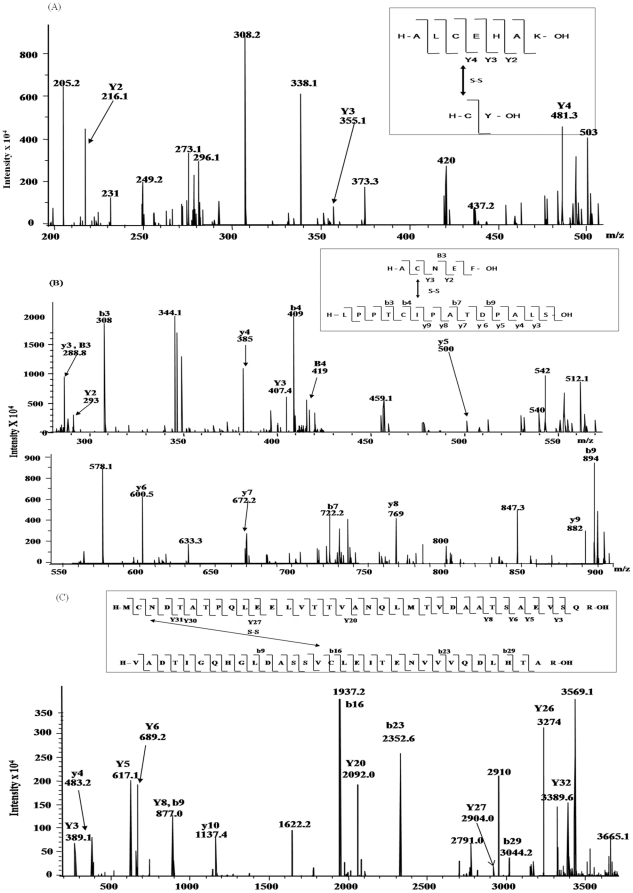
LC-ESI-MS/MS of disulfide connected dipeptide. (A) LC-ESI-MS/MS *m/z* 3566.5 (doubly charge), containing disulfide bonded Cys^2^-Cys^479^ peptide from trypsin and chymotrypsin digested MtbDGC. Inset shows the sequence derived from this MS/MS spectrum. (B) LC-ESI-MS/MS of *m/z* 1053.2, containing disulfide bonded Cys^94^-Cys^584^ peptide from trypsin and chymotrypsin digested intact unreduced MtbDGC. Inset shows the sequence derived from this MS/MS spectrum. (C) LC-ESI-MS/MS of *m/z* 2799.1, containing disulfide bonded Cys^429^-Cys^614^ peptide from trypsin and chymotrypsin digested MtbDGC. Inset shows the sequence derived from this MS/MS spectrum.

MS/MS analysis of the doubly charged [M +2H]^2+^ ion at *m/z* 3566.5, obtained from chymotryptic and tryptic digested sample, alkylated with iodoacetamide, confirmed that the peptide containing Cys^2^ (*m/z* 3739.2) was linked to the peptide containing Cys^479^ (*m/z* 3391.8) (Figure 9A). The CID spectrum of dipeptide containing Cys^2^ and Cys^479^ showed successive b and y type ions of *m/z* 389.1 (Y3), 483.4 (y4), 617.7 (Y5), 689.2 (y_6_), 877.0 (y8, b9), 1137.4 (y10), 1937.2 (b16), 2092.0 (Y20), 2352.6 (b23), 2904.0 (Y27), 3044.2 (b29), 3274 (y26) and 3389.6 (Y32). Evidence was also obtained for another expected disulfide pair (Cys^94^-Cys^584^). As shown in Figure 9B, the MS/MS of singly charged ion at *m/z* 1053.2 confirms that peptide containing Cys^94^ (*m/z* 770) is linked with peptide containing Cys^584^ (*m/z* 284.3). The CID spectrum showed successive b and y type ions of *m/z* 288.8 (B3), 293 (Y2), 407.2 (Y3), 308 (b3), 409 (b4), 722.2 (b7), 894 (b9), 288.8 (y3), 385 (y4), 500 (y5), 600.5 (y6), 672.2 (y7), 769 (y8) and 882 (y9). As shown in Figure 9C, the tandem mass spectra obtained from the ion *m/z* 1976.8 confirm the structure of peptide containing the third disulfide bridge between Cys^429^-Cys^614^. Figure 9C shows that the prominent fragments are derived from the singly charged ion of dipeptide containing Cys^429^ and Cys^614^. From MS/MS analysis it can be seen that only minor fragmentation occurred within the amino acid sequence between disulfide linked cysteine residues. The data showed no other disulfide linked peptides, other than the expected. It is consistent with the fact that it is difficult to cleave peptides that contain –S-S- bonds as fragmentation requires cleavage of double bonds [28]. No evidence was found for the fragmentation of the amide bonds and formation of linearized sequences by cleavage of peptide bonds inside the disulfide ring [50].

Similar dissociation fragmentation processes have already been reported earlier for intramolecular disulfide-bonded peptides [26]. The obtained data confirmed the disulfide connectivity within intramolecular disulfide bond, but not intermolecular disulfide bond. Similar results to those shown in Figure 9(A–C) were observed for chymotrypsin and trypsin digested samples treated with or without iodoacetamide.

### Sequence alignment of proteins containing GAF, GGDEF, EAL domains and homology modeling of Rv1354c

**Figure 10:**
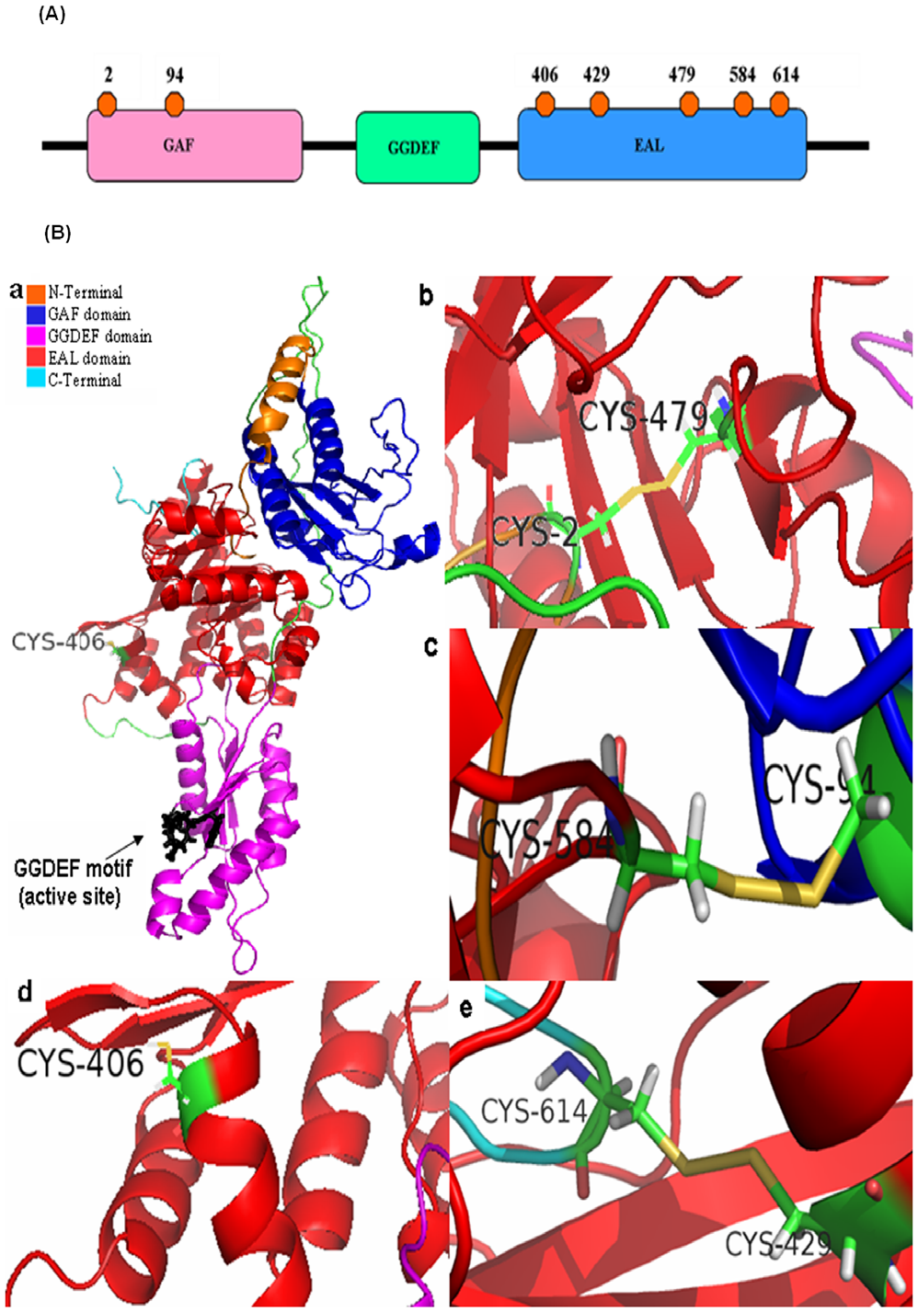
Modeled MtbDGC. (A) Distribution of seven cysteines on full length MtbDGC. (B) Modeled MtbDGC with the disulfide bonds. (a) Modeled protein with exposed Cys406 residue. GGDEF motif (residue number 261–265) and is shown in black color. (b) Disulfide bond between Cys residue Cys2-Cys479 (c) Disulfide bond between Cys residue 894-584. (d) free and exposed Cys406 (e) disulfide between Cys residue 429-614.

There is no structure available for full length GAF-GGDEF-EAL bifunctional protein. However, to probe the disulfide connectivity, even an approximate structure would be very useful. Structure obtained by modeling studies (Figure 10) showed a good correspondence between the predicted disulfide connectivity obtained by mass spectral analysis.

**Figure 11:**
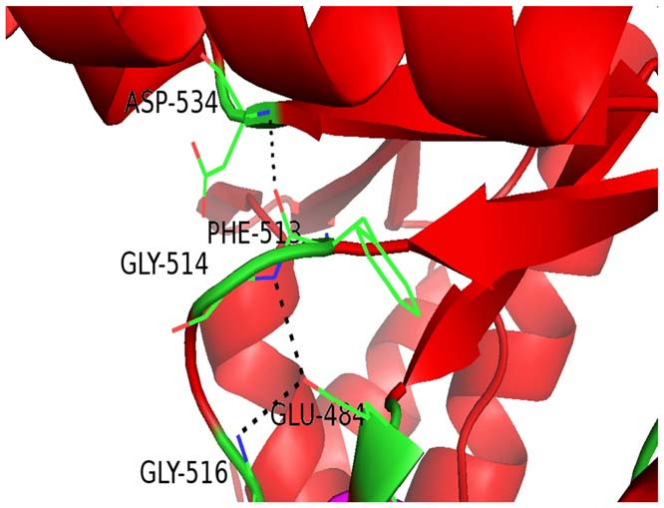
Part of EAL domain with the residues of loop 6. Hydrogen bond between residues is shown in the black broken line.

**Figure 12:**
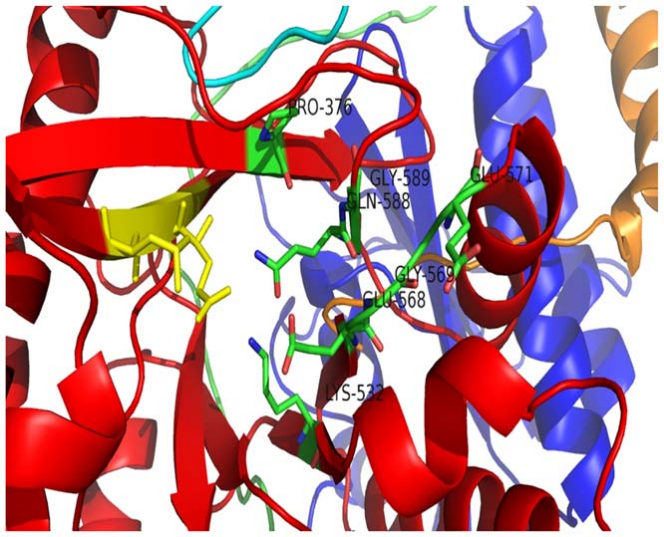
EAL motif shown in yellow color and the conserved residues in close proximity to the motif is highlighted.

Schmidt and coworkers [51] first noticed that the catalytically active EAL domains seem to contain a conserved motif that was later confirmed to contain loop 6 [DFG(T/A)GYSS], loop between the α6-helix and β6-strand and one of the residues (Asp) for Mg^2+^ binding [51], [52]. Alignment of the EAL domain sequences shows a conserved motif [DFG(A/S/T)(A/G)(Y/F)(S/T)(S/T/G/A/N)] or also called loop 6 (Figure S1), apart from conserved EAL motif and many conserved acidic residues. Similar result was found when EAL domain (PF00563) family (total of 8082 sequences) in PFAM database, was analyzed (data not shown). Out of three domains named as GAF (residue 28–171), GGDEF (residue 212–345) and EAL (residue 354–609), EAL domain is the biggest with (β/α)_10_ barrel fold with 10 α-helix and β-strands each assigned by STRIDE [53]. Percentage consensus of G^2^, G^4^, F^3^, S^7^ and S^8^ in the loop 6 was found to be 88%, 79%, 80%, 65% and 61% respectively highlighting the importance of both Glycine residues along with Phenylalanine in the loop. Above result is based on the alignment of 8082 sequences belonging to EAL domain family. G^514^ and G^516^ are involved in the side chain - main chain hydrogen bonding with E^484^, while F^513^ and D^534^ residues are involved in the main chain-main chain hydrogen bonding (Figure 11) [54]. Hence, it can be concluded that the above three residues are important for the positioning of the loop 6. Depending upon the involvement of residues of loop 6 in forming hydrogen bond, they can be divided into two parts: i) D^512^F^513^G^514^T^515^G^516^ (ii) Y^517^S^518^A^519^. The first half gives stability and proper positioning to the loop, while the latter half is flexible. Alignment of EAL domain sequences obtained from the GAF, GGDEF, EAL architecture shows very high conservancy of P^376^, E^484^, G^505,^ L^523^, K^532^, E^568^, G^569^, V^570^, E^571^,Q^588^G^589^. As discussed earlier E^484^ is involved in the hydrogen bonding with two glycines of loop 6. On mapping P^376^, K^532^, E^568^, G^569^,E^571^, Q^588^, G^589^ on the modeled structure, it was found that these residues are in close proximity with E^389^A^390^ L^391^ motif (Figure 12) may also play an important role in coordinating with Mg^2+^ions.

GGDEF domain sequences were also aligned in the same fashion as the EAL domain sequences and were found to have high conservancy of G^232^, D^237^, G^307^ & R^259^ apart from GGDEF^261–265^ motif (Figure S2). When the above three residues were mapped on the structure, D^237^ is found to be on the surface, possibly making it important in oligomerization. High conservancy of G232 can be related with the need of neglecting satiric clashes and the proper orientation of the two consecutive helices. R^259^ may help in the stabilizing the GTP-GGDEF domain complex as it is close to the GGDEF motif.

P^131^ & G^140^ of GAF domain shows high conservancy (Figure S3). Both of them are part of beta strands and at the same time they are involved in van der Waals interaction with R^381^ and N^54^ respectively.

Although the part of linker region between GAF and GGDEF domain (linker 1; residue 184–211) is considered as a part of GGDEF domain, yet while analyzing it was considered as a part of linker region (according to SWISSPROT linker 1 ranges from residue 172–211) and analysis shows the high conservancy of D^187^, T^190^ & N^194^, R^195^(Figure S4). All are polar residues and may play important role in stabilizing the oligomer of the proteins.

Several N-terminal residues (residue 1–28) are involved in either main chain-main chain (M^1^-V^565^, C2-V^565^,R^583^-D^4^,A^6^-D^581^) or side chain-main chain (T^7^-T^165^,Q^9^-A^159^) interaction with the residues of GAF and EAL domain. At the same time C^2^ forms a disulfide bond with C^479^, showing the importance of the N-terminal residues in stabilizing the GAF-EAL interface. Although C-terminal residues are not involved in the formation of hydrogen bond, C^614^ forms a disulfide bond with C^429^. Hydrogen bonds were found between the GAF and EAL domain residues also (data not shown). Interestingly enough GAF domain does not interact with the GGDEF domain, but interactions were found between the linker 1(residue 172–211).

EAL-GGDEF interface has lesser number of interactions compared to that of the GAF-EAL interface. Few main chain-main chain & side chain-main chain hydrogen bonds were found (data not shown).

Van der Waals interaction between two atoms was evaluated using CONTACT program of CCP4 program suit by setting the limit of distance below 4.5 Å [55]. The result shows that although EAL is the last domain, yet it is in contact with both GAF and GGDEF domains.

The proposed structure of Rv 1354c protein shows well exposed GGDEF motif. Disulfide bond length was found to be 2.3 A° (Cys^2^-Cys^479^, Cys^429^-Cys^614^) and 2.2 A° (Cys^94^-Cys^584^). Cys^406^ residue of EAL domain is more solvent assessible than other six cysteine residues.

### Construction of Cysteine mutant by site directed mutagenesis

**Figure 13:**
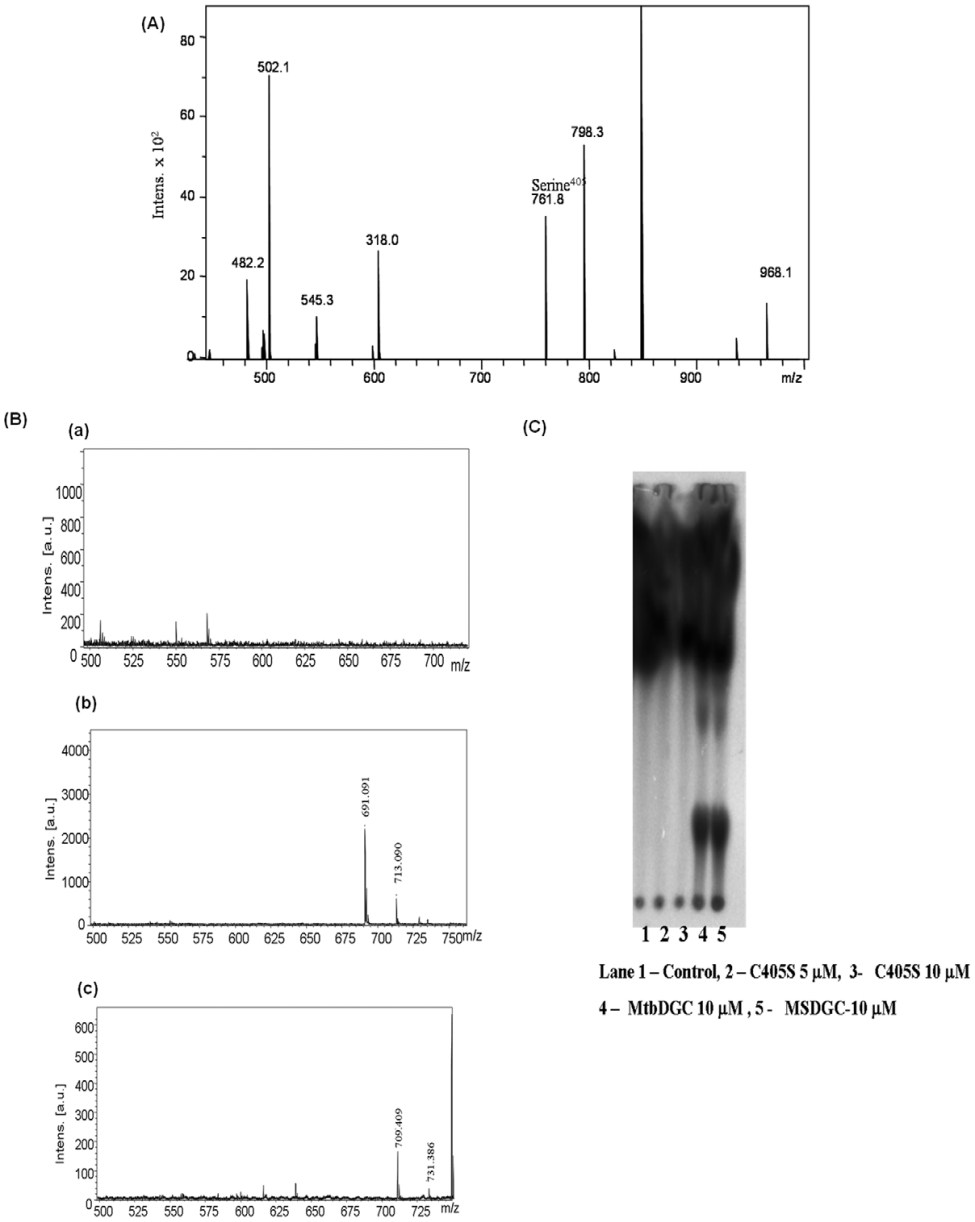
Functional activity of MtbC406S mutated protein. (A) LC-ESI-MS of DTT reduced MtbC406S digested with trypsin and chymotrypsin. The *m/z* 760.9 shows the substitution of Cys^406^ to Serine^406^. (B) (a) MtbC406S protein: MALDI TOF analysis, in the positive ion detection mode. No peaks are obtained at 691 (M+H)^+^ and 713 (M+Na)^+^ for c-di-GMP, 709 (M+H)^+^ and 731 (M + Na)^+^ for pGpG (b) MtbDGC protein for control: MALDI TOF analysis, in the positive ion detection mode, ions were detected at 691 (M+H)^+^ and 713 (M+Na)^+^ for c-di-GMP (c) 709 (M+H)^+^ and 731 (M + Na)^+^ for pGpG.

**Figure 14:**
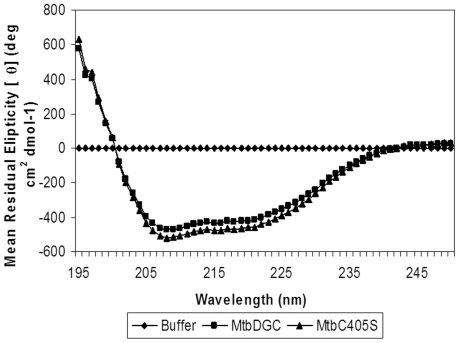
Circular Dichorism spectra of MtbDGC and MtbC406S.

From the Mass spectral data it was confirmed that cysteine at position three (Cys^406^) is free. To further elucidate whether Cys^406^ has any role in the enzymatic activity of the protein, the Cys^406^ was mutated to serine. Cysteine mutation was confirmed by LC-MS and the mutated protein showed the mass of 68459.93 Da (histidine tagged) (data not shown). MtbC406S was further reduced with 8 mM of DTT and the reduced protein showed the mass difference of seven Da confirming the presence of only three cysteine bond and Cys^3^ (Cys^406^) is free (data not shown). The chymotrypin and trypsin digested MtbC406S shows the change of 16 Da in the peptide bearing cysteine at position three confirming the mutation of Cys^406^ to Serine^406^ (aa. 400–407) (Figure 13A). MS/MS analysis of mutated peptide (aa. 400–407) obtained from trypsin and chymotrypsin digested protein showed the mutation of cysteine to serine (data not shown). In order to check the activity of c-di-GMP in MtbC406S protein, the mass spectrometric as well as thin layer chromatographic analysis were done as shown in Figure 13B and Figure 13C. It is clear from the data that Cys^406^ which is a free cysteine plays a critical role in the regulation of enzymatic activity and by mutating cysteine to serine, protein activity is lost. However, when CD spectrum of the mutated protein was compared with MtbDGC protein (Figure 14) and no difference was found in the folding pattern of the protein. This indicates that Cys^406^ plays an important role in c-di-GMP regulation but does not play any significant role in the folding of the protein.

### Concluding remarks

The cyclic dinucleotide 3′, 5′ cyclic guanylic acid (c-di-GMP) has been characterized as an important second messenger that affects a range of physiological traits in bacterial species. Many reports have come in recent past, from diverse branches of bacteria utilizing c-di-GMP signaling pathways [56], [57]. Prior to our study in *Mycobacterium smegmatis* (Kumar and Chatterji, 2008), nothing was known about the role of c-di-GMP in Mycobacteria. We reported that MSDGC-1 protein is a bifunctional protein and can synthesize and degrade c-di-GMP *in vitro*. This study was undertaken to follow the role of C-di-GMP in *M. tuberculosis*. In this work we investigate the homologue of MSDGC-1, MtbDGC in *M. tuberculosis* H37Rv. We reveal the following findings: (1). *M. tuberculosis* has one protein with GAF-GGDEF-EAL (MtbDGC) and another with only EAL domain (MtbPDE). (2). MtbDGC is a bifunctional multidomain protein exhibiting both the activity of DGC and PDE-A. (3). MtbPDE is a single EAL protein exhibiting only PDE-A activity. (4) Presence of three disulfide bond and one free cysteine in MtbDGC protein. (5) Upon mutation of free cysteine at position 406 to serine, synthesis of C-di-GMP was completely abolished.

All the known examples of GGDEF-EAL domain either have DGC or PDE-A activity. However, it is difficult to predict the dominant activity from the primary sequence [56], [58]. In *Rhodobacter sphaeroides* and *Vibrio parahaemolyticuss* GGDEF/EAL protein shows both DGC and PDE-A activities as a function of environmental cue [59], [60], but no clear cut distinction of the activities were possible. In this regard mycobacterial protein (MSDGC-1 and MtbDGC) is the first such protein which shows two activities at a time *in vitro*. It appears that both *M. tuberculosis* and *M. smegmatis* proteins have similar function; the role of additional EAL domain protein in *M. tuberculosis* is yet to be addressed.

*M. tuberculosis* DGC protein reported here was found to be predominantly expressed at the stationary phase suggesting its role in survival at stationary phase. This hypothesis was substantiated by complementation of MSDGC-1 knockout strain of *M. smegmatis* by mtbdgc. From sequence analysis and presence of dual activities it appears that both *M. smegmatis* and *M. tuberculosis* DGC will have similar function in the respective host. However, the only difference is presence of seven cysteines in MtbDGC. Comparison of the sequences of DGC protein revealed that three cysteines are conserved in MSDGC-1 out of seven in MtbDGC. It is interesting to study the presence of high numbers of cysteines in MtbDGC with regards to folding and regulation of c-di-GMP signaling at enzymatic level.

In order to address above hypothesis we studied disulfide bonded cysteine and free cysteine in MtbDGC. Disulfide bonded cysteine might play and crucial role in folding of MtbDGC, whereas free cysteine may be involved in regulation activity of enzyme. Extensive mass spectrometric analysis confirmed the presence of three disulfide bonded Cys^94^-Cys^584^, Cys^2^-Cys^479^ and Cys^428^-Cys^614^ and one free cysteine at position Cys^406^. Data obtained from mass spectrometric analysis was further confirmed by homology modeling.

Structure obtained by homology modeling revealed presence of three disulfide bonds and one free cysteine with exposed GGDEF motif. The homology model presented here, is the first example which shows that the last (EAL) domain comes in the middle interacting with the other two (GAF & GGDEF) domains. Probably it may hint in the possible mechanism regulating the activities of such proteins having both GGDEF & EAL domains to avoid a possible futile cycle. Apart from conserved motifs and polar residues, significant number of conserved Glycine residues was also found. Almost all conserved residues are either involved in the van der Waals interaction or in the hydrogen-bonding and are important in maintaining the orientation of the domains and thereby the shape of the protein.

Cys406 was found to be distinct far from the remaining six cysteines and does not participate in any disulfide connectivity. As we hypothesized that free cysteine might have regulatory role. We mutated Cys^406^ to Serine and enzymatic activity was assayed. C-di-GMP synthesis activity was completely abolished in mutated protein. The exact role of Cys^406^ in mechanism of c-di-GMP synthesis still remains to be investigated. To our best knowledge this is the first example of regulation of enzymatic activity by free cysteine in GAF-GGDEF-EAL protein.

## Supporting Information

Figure S1 **Alignment of EAL domain from the protein having GAF, GGDEF, EAL architecture.** Protein of our interest is highlighted and [DFG(A/S/T)(A/G)(Y/F)(S/T)(S/T/G/A/N)] motif is shown in the box. Protein of interest contains DFGTGYSA motif.
(TIF)

Figure S2 **Alignment of GGDEF domain from the protein having GAF, GGDEF, EAL architecture.** Protein of our interest is highlighted and G232, D^237^, G^307^, R^259^ & GGDEF^261-265^ motif is shown in box. Residue number of residues corresponds to the number in the protein chosen.
(TIF)

Figure S3 **Alignment of GAF domain from the protein having GAF, GGDEF, EAL architecture.** Protein of our interest is highlighted and P^131^ & G^140^ are boxed.
(TIF)

Figure S4 **Alignment of GGDEF domain from the protein having GAF, GGDEF, EAL architecture.** Protein of our interest is highlighted and D^187^, T^190^ & N^194^R^195^ are shown in box.
(TIF)
