# Supplementary figures and images for: Identification, Activity and Disulfide Connectivity of C-di-GMP Regulating Proteins in Mycobacterium tuberculosis
Source: PLoS One. 2010 Nov 30;5(11):e15072. doi: 10.1371/journal.pone.0015072 (PMC2994820; doi:10.1371/journal.pone.0015072)

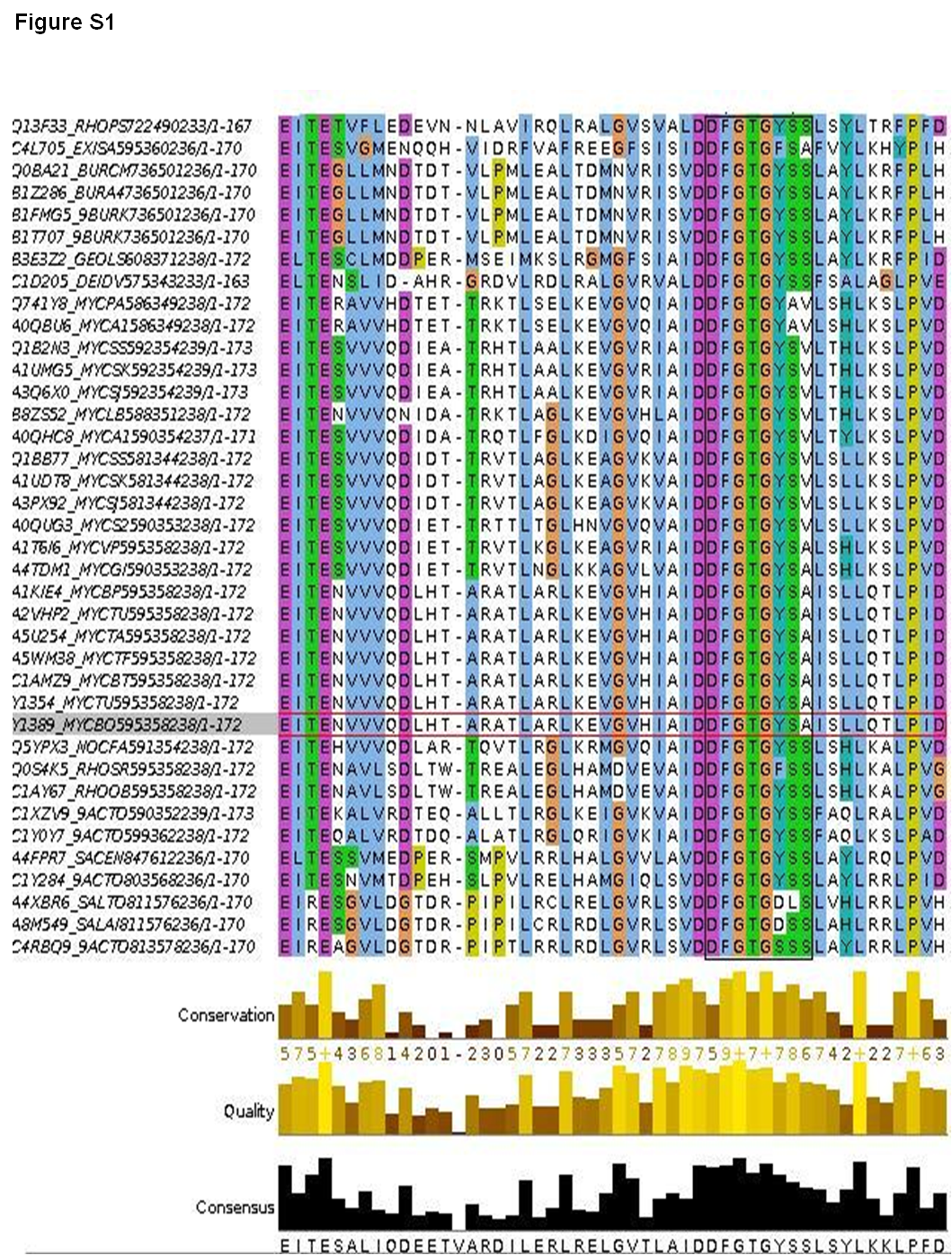

Supplement: Figure S1 — Alignment of EAL domain from the protein having GAF, GGDEF, EAL architecture. Protein of our interest is highlighted and [DFG(A/S/T)(A/G)(Y/F)(S/T)(S/T/G/A/N)] motif is shown in the box. Protein of interest contains DFGTGYSA motif. (TIF) [file pone.0015072.s001.tif]

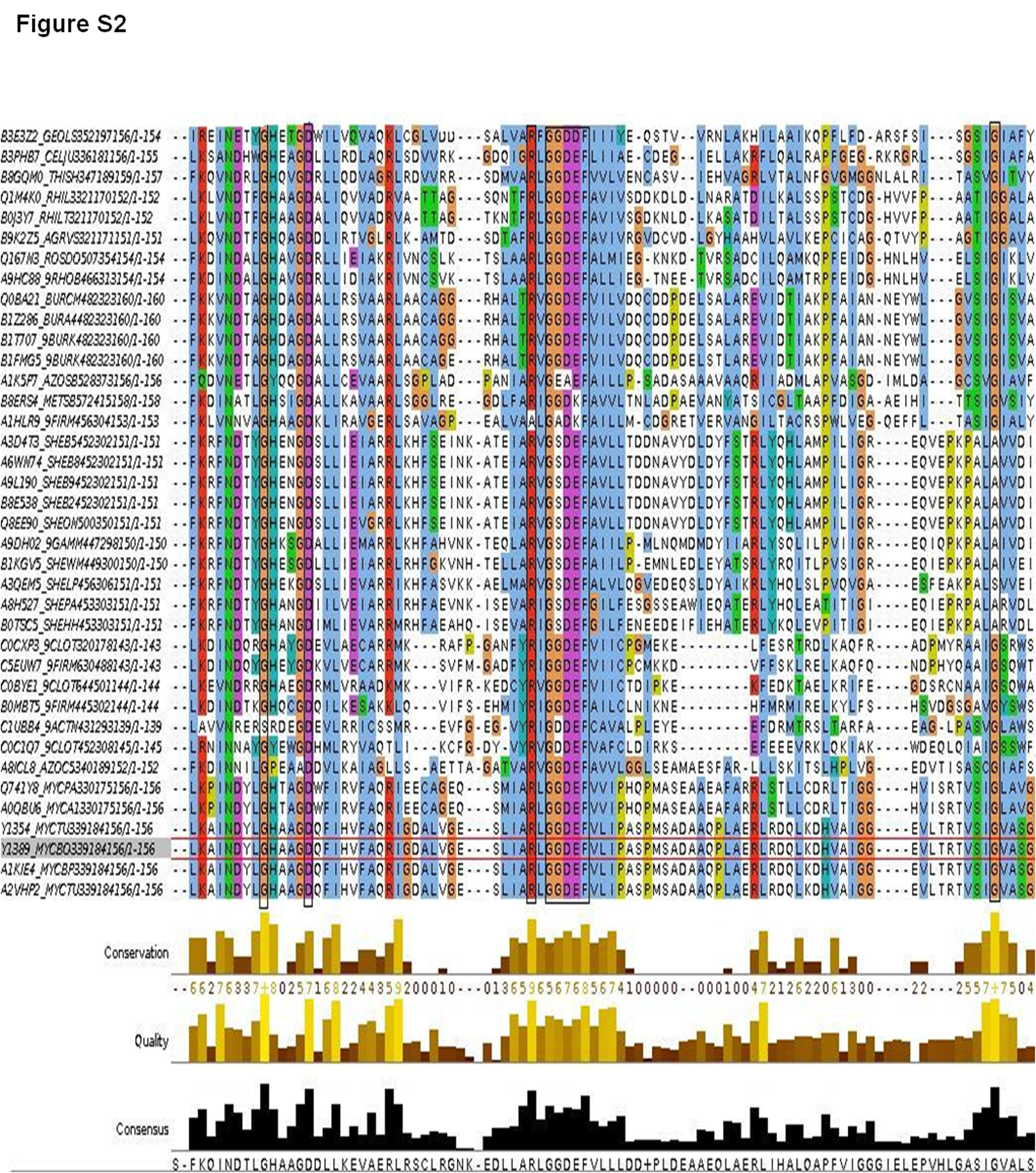

Supplement: Figure S2 — Alignment of GGDEF domain from the protein having GAF, GGDEF, EAL architecture. Protein of our interest is highlighted and G232, D237, G307, R259 & GGDEF261-265 motif is shown in box. Residue number of residues corresponds to the number in the protein chosen. (TIF) [file pone.0015072.s002.tif]

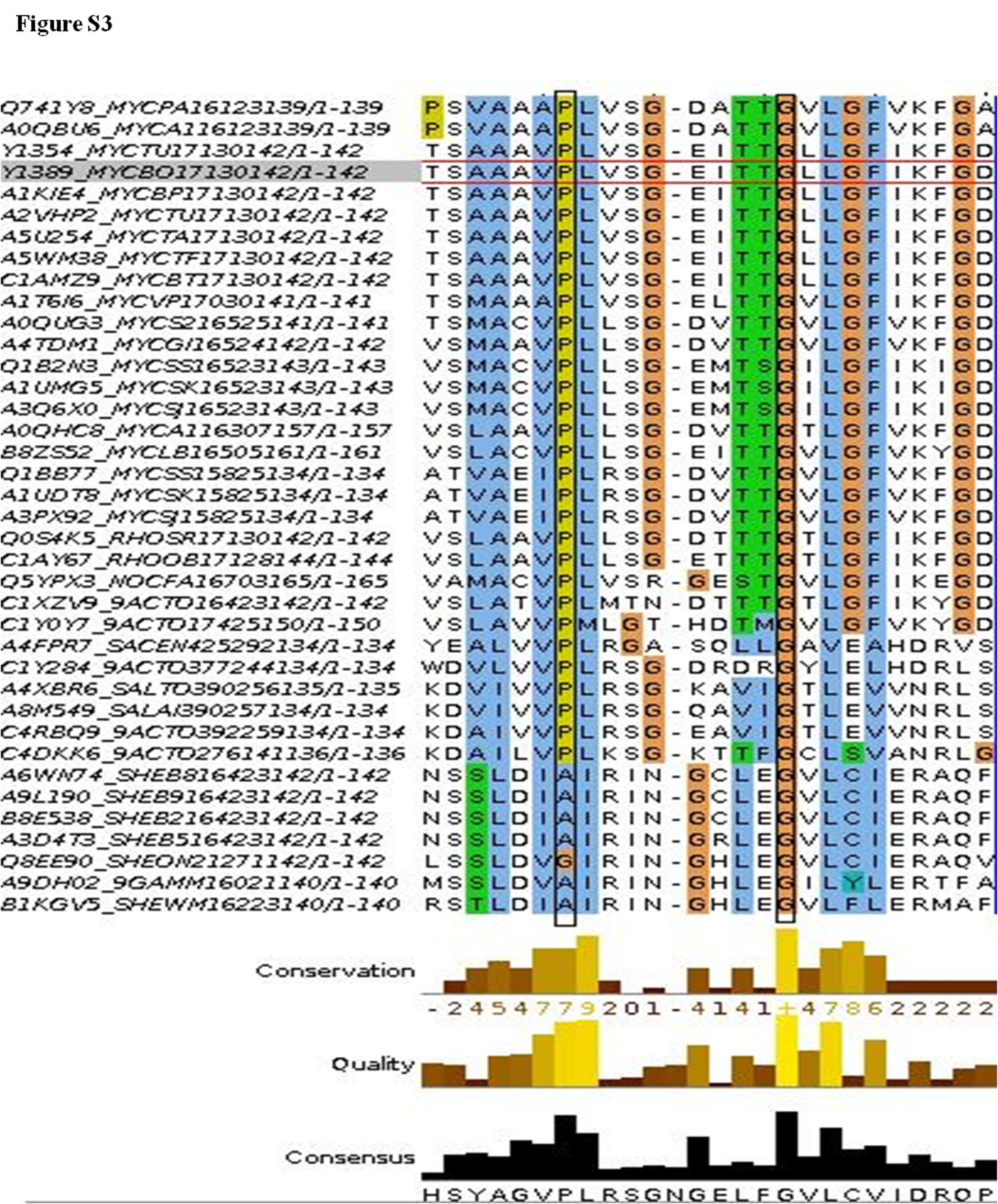

Supplement: Figure S3 — Alignment of GAF domain from the protein having GAF, GGDEF, EAL architecture. Protein of our interest is highlighted and P131 & G140 are boxed. (TIF) [file pone.0015072.s003.tif]

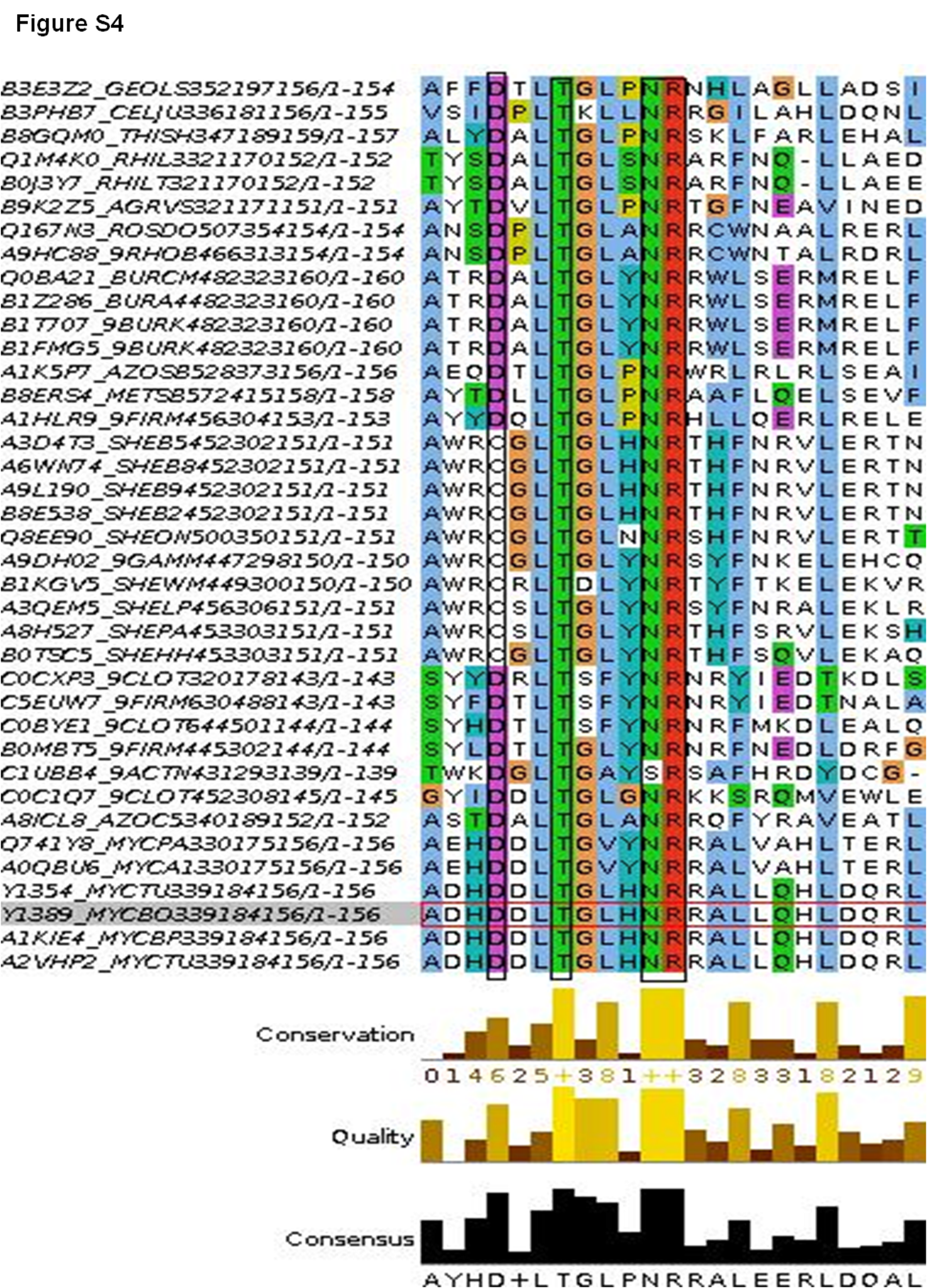

Supplement: Figure S4 — Alignment of GGDEF domain from the protein having GAF, GGDEF, EAL architecture. Protein of our interest is highlighted and D187, T190 & N194R195 are shown in box. (TIF) [file pone.0015072.s004.tif]
